# Transumbilical Single-Incision Laparoscopic-Assisted Appendectomy (TULAA) is Useful in Adults and Young Adolescents: Comparison with Multi-Port Laparoscopic Appendectomy

**DOI:** 10.3390/medicina55060248

**Published:** 2019-06-05

**Authors:** Seung Gyu Jin, Seong Hoon Cho, Kwang Yong Kim, Soo Kyung Ahn, Ji Woong Hwang, Ji Woong Cho, Bong Wha Jung, Byung Chun Kim, Sang Nam Yoon

**Affiliations:** Department of Surgery, Kangnam Sacred Heart Hospital, Hallym University College of Medicine, Seoul 07441, Korea; jinplem@hallym.or.kr (S.G.J.); csh1989@hallym.or.kr (S.H.C.); kyoung79@hallym.or.kr (K.Y.K.); ahnsookyung@hallym.or.kr (S.K.A.); dattoree@hallym.or.kr (J.W.H.); jwcho@hallym.or.kr (J.W.C.); bkys831@hallym.or.kr (B.W.J.); bckimgs@hallym.or.kr (B.C.K.)

**Keywords:** appendicitis, appendectomy, transumbilical single-port laparoscopic-assisted appendectomy, laparoscopic appendectomy, interval appendectomy

## Abstract

*Background and objectives:* Single-port laparoscopic appendectomy (SLA) in most previous studies has used intracorporeal excision of the appendix and needed a longer operative time than multi-port laparoscopic appendectomy (MLA), although SLA does have the potential benefit of an almost invisible scar within the umbilicus. Some studies have reported that extracorporeal transumbilical single-incision laparoscopic-assisted appendectomy (TULAA) in children took a considerably reduced operative time compared to MLA. We adopted TULAA in adults, adding routine dissection of the peritoneal attachment of the appendix. The aim was to compare the operative outcomes between TULAA and MLA. *Materials and Methods:* Between March 2013 and January 2016, 770 patients with acute uncomplicated and complicated appendicitis from 15 to 75 years of age were enrolled retrospectively. The operation was performed as early (EA) and interval appendectomy (IA). *Results:* Operative time was shorter in the TULAA group than in the MLA group, except for IA. No open conversion occurred in the TULAA group, except one case of ileocecal resection for IA. No intra-abdominal fluid collection was found in the TULAA group. Extended resection (especially partial cecectomy) was performed less frequently in the TULAA group than in the MLA group for IA. Mean postoperative hospital stay was shorter in the TULAA group for uncomplicated appendicitis. When the data of the EA group and the IA group were compared, operative time was significantly shorter in the IA group for both MLA and TULAA. The open conversion rate and the complication rate tended to be lower in the IA group. Confined to IA, the TULAA group tended to have shorter mean initial, postoperative, and total hospital stays. *Conclusions:* TULAA can be a useful surgical alternative to MLA in adults and young adolescents, because it lacks open conversion and provides both a shorter operative time and a shorter postoperative hospital stay. TULAA is feasible for IA in that it showed a lower rate of extended resection and complications.

## 1. Introduction

As a minimally invasive surgery, laparoscopic appendectomy yields favorable outcomes and better cosmetic results for acute appendicitis than open appendectomy [1,2]. Single-port laparoscopy has been proposed as an alternative to multi-port laparoscopy in a few surgical procedures, including appendectomy [3] and cholecystectomy [4], as well as for the management of colorectal neoplasm [5,6]. Previous studies compared single-port laparoscopic appendectomy (SLA) and multi-port laparoscopic appendectomy (MLA), and most of them concluded that SLA is not superior to MLA because SLA is associated with a longer operative time and yields similar cosmetic results [7,8,9,10,11,12].

A few pediatric surgeons have attempted ‘extracorporeal’ resection of the appendix via single umbilical incision and have usually called it transumbilical single-incision laparoscopic assisted appendectomy (TULAA) [13,14,15,16,17]. Recent studies about TULAA show a shorter operative time than for conventional MLA [18,19,20,21]. We introduced a similar surgical technique for adults and young adolescents with a few modifications. We used commercial single-port devices and routinely dissected the peritoneal attachments to the appendix and cecum to exteriorize the appendix easily via the transumbilical incision.

Early appendectomy (EA) for complicated appendicitis is known to have an increased risk of extended resections (partial cecectomy or ileocecal resection) and postoperative complications (wound problems, ileus, intra-abdominal fluid collection, etc.) [22,23]. It has been suggested that interval appendectomy (IA) could reduce the problems associated with EA. IA is recommended six to eight weeks after appendicitis has developed because it can prevent the recurrence of appendicitis, and exclude a diagnosis of neoplasms, such as carcinoid, adenocarcinoma, mucinous cystadenoma, or cystadenocarcinoma [24,25]

We compared operative outcomes between the TULAA group and the MLA group for uncomplicated and complicated appendicitis in adults and young adolescents. In addition, we aimed to assess the feasibility of TULAA applied to IA for complicated appendicitis.

## 2. Materials and Methods

### 2.1. Patients

**Figure 1:**
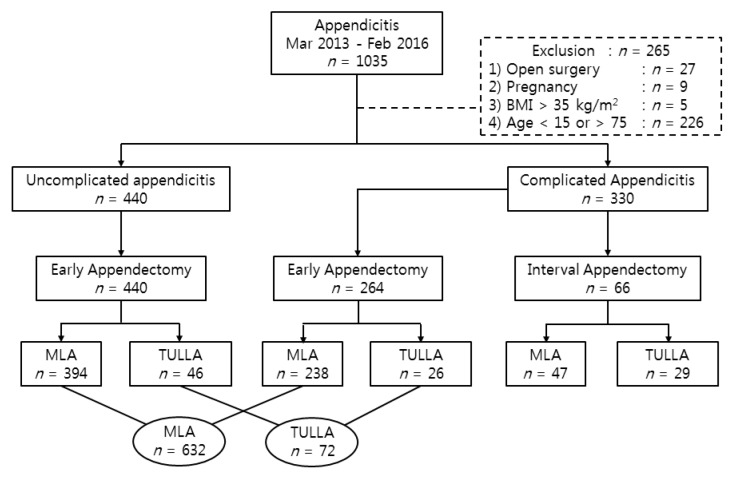
Flow sheet of total patients. BMI, body mass index (kg/m^2^); MLA: multi-port laparoscopic appendectomy; TULAA: transumbilical single-incision laparoscopic-assisted appendectomy.

Between March 2013 and February 2016, 1035 patients in Kangnam Sacred Heart Hospital, Seoul, Republic of Korea, who had undergone surgery, including TULAA and MLA, for acute appendicitis were surveyed in this study retrospectively. Diagnosis of appendicitis was based on clinical suspicion, laboratory parameters, and imaging studies. The majority of the patients had received computed tomography, and a few had had ultrasonography. Uncomplicated appendicitis was defined as lacking appendiceal perforation and/or periappendiceal abscess formation. The patients were classified as having complicated appendicitis if the computed tomography (CT) results showed periappendiceal abscess, phlegmon, or perforation. Patients who were excluded were those with open appendectomy (*n* = 25), pregnancy (*n* = 9), body mass index (BMI) (kg/m^2^) >35 (*n* = 5), or ages younger than 15 years (*n* = 214) or older than 75 years (*n* = 12). Finally, 440 patients with uncomplicated appendicitis and 330 patients with complicated appendicitis were enrolled. A flow sheet of the total patients is shown in Figure 1. The authors analyzed the patients with high heterogeneity by dividing them into several subgroups. The study was approved by the Institutional Review Board of the Kangnam Sacred Heart Hospital (Seoul, Korea) (IRB number: 2016-12-159, approved on: 21 December 2016). Informed consent was obtained from all patients.

### 2.2. Interval Appendectomy

All patients with uncomplicated appendicitis underwent EA. Patients with complicated appendicitis had either EA or IA according to the surgeons’ preferences. Patients who were to undergo IA were treated conservatively at the initial presentation, and then underwent the operation six to eight weeks later.

Intravenous (IV) antibiotics were administered during initial hospitalization and oral antibiotics were prescribed after discharge. The most commonly used IV antibiotics were a combination of second-generation cephalosporin (cefuroxime) and metronidazole. Third-generation cephalosporin (ceftriaxone or cefotaxime) was used for cases with severe inflammation. If a patient was allergic to cephalosporin antibiotics, ciprofloxacin was administered. Prescribed oral antibiotics were either second generation (cefaclor) or third generation (cefditoren) cephalosporin. Occasionally, metronidazole was added if needed. Total duration of antibiotic use was generally two weeks but sometimes more.

A percutaneous catheter drainage (PCD) was inserted if the initial or repeated CT showed a gross abscess around the appendix. We decided to repeat the CT if a patient showed a persistent fever, tenderness, and leukocytosis for longer than three days after admission despite intravenous antibiotics.

### 2.3. Preoperative Preparation

Intravenous (IV) antibiotics were administered during hospitalization and oral antibiotics were prescribed after discharge for two days. Preoperative IV antibiotics were injected within an hour before a skin incision. Injected IV antibiotics were the same as above. Suprapubic hair was shaved with a razor blade before operation. No nasogastric tube was inserted. No Foley catheter was introduced for uncomplicated acute appendicitis unless appendiceal perforation was detected during the operation.

### 2.4. Operative Technique

The surgeons decided on the type of operation according to their own preferences. Seven surgeons usually performed MLA, and one surgeon performed TULAA. All eight surgeons were regular staff members and had done more than 50 MLAs.

**Figure 2:**
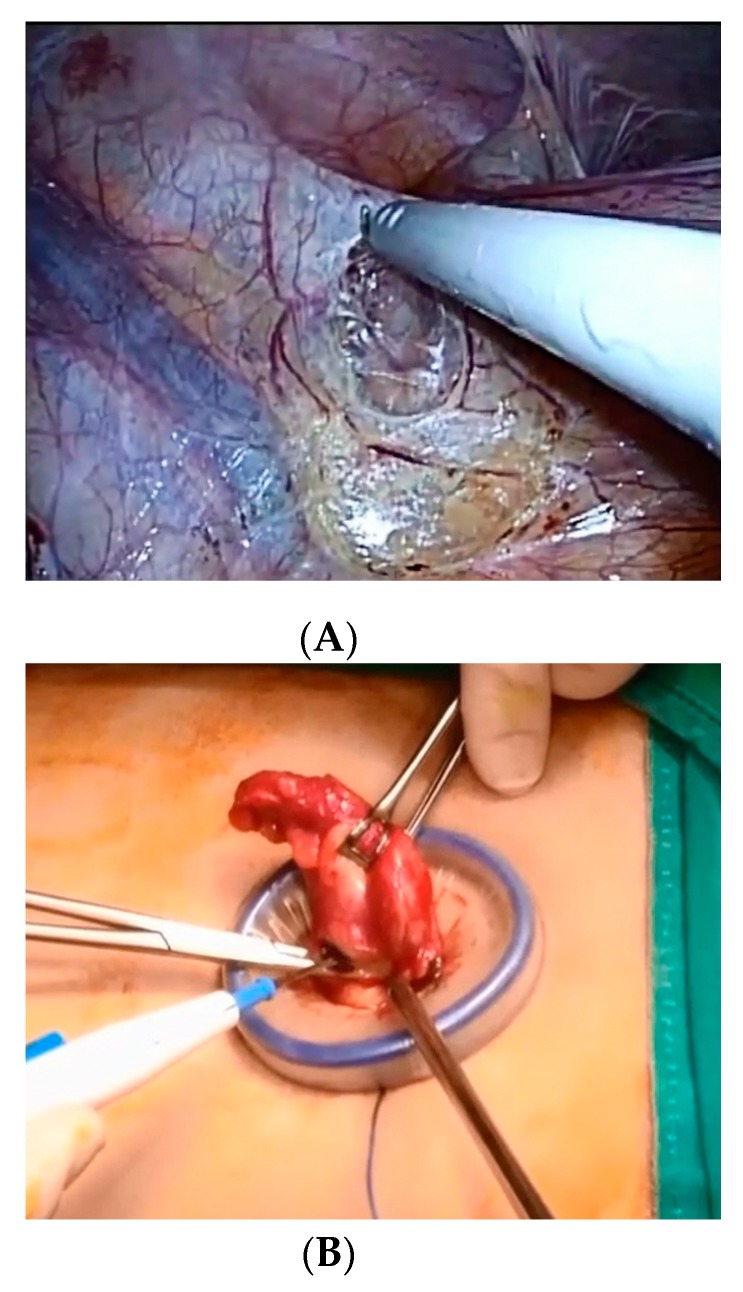
Transumbilical single-incision laparoscopic-assisted appendectomy. (**A**) The peritoneal attachments to the appendix and cecum were dissected by pulling the appendix to the cephalo-medial direction. (**B**) The mesoappendix was clamped and divided after the appendix was removed through the transumbilical incision protected by the wound protector.

For TULAA, patients were positioned supine with the left arm tucked and a general anesthetic was administered. The umbilicus was cleansed from dirt with a cotton swab and sterilized using 70% isopropanol before preparation of the skin with iodine solution. A 1.5 cm vertical transumbilical incision was made within the umbilical dimpling with traction using Allis forceps grasping each side of the umbilicus. Subcutaneous fat and fascia were dissected to allow entry and direct vision into the peritoneal cavity. Fascia was incised wider than the skin incision. A single-port device composed of a wound protector and a cap with trocars (Lap Single^®^ (Sejong Medical, Paju, Gyeonggi-do, Korea) or (Glove Port^®^ (Nelis, Bucheon, Gyeonggi-do, Korea)) was applied to the incision site, and CO_2_ was used to inflate the peritoneal cavity. An operating table was tilted to the Trendelenburg position and rotated to the left side. The instruments used were a 10 mm, 30° rigid scope for inspection, a standard 5 mm grasper for holding organs, and an ENDOPATH electrosurgery Probe Plus II system with a hook electrode (Ethicon, Somerville, NJ, USA) for dissection, irrigation, and suction. After exploration of the peritoneal cavity, the peritoneal attachments to the appendix and cecum were dissected using electrocautery to allow the appendix to reach the umbilicus (Figure 2A). The appendix was clamped with a grasper, and the peritoneal cavity was deflated. A cap of the single port device was opened, and a wound protector in the single-port device was left at the transumbilical incision site. The appendix was extricated through the transumbilical incision by pulling out the grasper holding the appendix. The mesoappendix was divided and ligated with 2-0 Silk (Figure 2B), and the appendix was ligated at its base with two 1-0 Silk and amputated above the ties by electrocautery. The stump was returned into the abdomen and any residual intra-abdominal fluid was aspirated with a suction tube, as is usually used for open surgery. The wound protector in the single-port device was removed, and the transumbilical incision was irrigated with normal saline. A draining tube was inserted for complicated appendicitis when the patient underwent EA. The fascial layer was closed with 2-0 Vicryl, and the dermis was sutured with 3-0 Vicryl in a deep dermal fashion.

For MLA, a 12-mm trocar for an optic scope was installed in the supra- or infraumbilical area, and pneumoperitoneum was established. Another two 5-mm trocars for laparoscopic instruments were inserted under laparoscopic vision in the left lower quadrant and the suprapubic area. A 10 mm, 0° rigid scope was used for intraperitoneal exploration. The mesoappendix was divided by an ultrasonic sealing device or electrocautery, and the appendix was ligated at its base with LapLoop® (Sejong Medical, Paju, Gyeonggi-do, Korea) and amputated above the ties via electrocautery. The resected appendix was transferred into a Lapbag® (Sejong Medical, Paju, Gyeonggi-do, Korea) and retrieved through the 12 mm periumbilical incision after the 12 mm trocar was removed. All incision sites were irrigated with normal saline. A draining tube was inserted for complicated appendicitis when they underwent EA. Only the fascial layer of the periumbilical incision was closed with 2-0 Vicryl, and all skin incisions were closed with 3-0 Nylon.

Open conversion was defined as the extension of a skin incision more than the designated incision to extract the resected specimen.

### 2.5. Postoperative Management

The patients were encouraged to walk in the corridor and to inhale deeply on the day of the operation. Abdominal pain was controlled using intramuscular NSAIDs. Oral intake of water was permitted a day or two after flatus passage and/or bowel sounds were detected. Oral intake of a soft diet was allowed once the patients were able to tolerate sips of water. The patients were discharged once postoperative pain was controlled by oral analgesics, no evidence of infectious complications were detected, and a soft diet was tolerated. Oral antibiotics, such as a second-generation (cefaclor) or a third-generation (cefditoren) cephalosporin, were prescribed for two days after discharge. Postoperative recovery of the patient was monitored and suture materials were removed a week after hospital discharge at an out-patient department.

### 2.6. Statistical Analysis

Fisher’s exact test was used for categorical variables. The Shapiro–Wilk test was performed to test the normality of continuous variables. Then, the independent *t*-test was used when data was normally distributed, and Mann–Whitney U test was used when data was not normally distributed. A two-tailed *p* < 0.05 was considered to be statistically significant. All calculations were performed using SPSS 23.0 version (IBM SPSS Statistics, IBM Corporation, Armonk, NY, USA).

## 3. Results

### 3.1. Comparisons between Multi-Port Laparoscopic Appendectomy and Transumbilical Single-Incision Laparoscopic-Assisted Appendectomy for Uncomplicated Appendicitis

Gender, age, and BMI were similar between the MLA group and the TULAA group (Table 1). Operative time and postoperative hospital stay were significantly shorter in the TULAA group than in the MLA group. No open conversion was detected in the TULAA group, whereas two open conversions occurred in the MLA group. The two open conversions were caused by small bowel adhesions traced to previous abdominal surgeries. No difference was found in the complications or re-admissions in either group. No intra-abdominal fluid collection was detected in the TULAA group, but six patients had postoperative intra-abdominal fluid collections in the MLA group; three of them required percutaneous drainage, and the other three recovered with antibiotics. No incisional hernia was found in either group during 39 months of median follow-up.

### 3.2. Comparisons between Early Appendectomy and Interval Appendectomy for Complicated Appendicitis

When the data was compared between the EA group and the IA group, the sex ratio and mean body mass index were similar in the two groups (Table 2). Mean age was higher in the IA group. A preoperative percutaneous catheter insertion was needed for gross abscess drainage in 18.2% of the patients in the IA group. TULAA was more frequently performed in the IA group. The need for extended resections and the open conversion rate were similar in the two groups. Mean operation time was significantly shorter in the IA group than in the EA group for both MLA and TULLA. The complication rate tended to be lower in the IA group than the EA group. In detail, for the complication rate, postoperative ileus tended to be more prevalent in the EA group, but wound infection and intra-abdominal fluid collection were similar in both groups. The leakage rates were too low to be interpreted. In the IA group, the postoperative hospital stay was significantly shorter, although the total hospital stay was higher than for the EA group. While 8% of the patients in the EA group were re-admitted, none of the patients in the IA group were re-admitted.

### 3.3. Comparisons between Multi-Port Laparoscopic Appendectomy and Transumbilical Single-Incision Laparoscopic-Assisted Appendectomy within the Early Appendectomy Group for Uncomplicated and Complicated Appendicitis

Gender, age, BMI, ratio of complicated appendicitis, complication rate, postoperative hospital stay, and re-admission rate were similar between the MLA group and the TULAA group (Table 3). There was a tendency for the rate of extended resections to be lower in the TULAA group, especially for partial cecectomy. Mean operative time was significantly shorter in the TULAA group. No open conversion was detected in the TULAA group, whereas 5.1% of patients were converted to open in the MLA group. No intra-abdominal fluid collection was detected in the TULAA group.

### 3.4. Comparisons between Multi-Port Laparoscopic Appendectomy and Transumbilical Single-Incision Laparoscopic-Assisted Appendectomy within the Interval Appendectomy Group for Complicated Appendicitis

Within the IA group, sex, mean age, and mean body mass index were similar between the MLA group and the TULAA group (Table 4). Initial, postoperative, and total hospital stays were significantly longer in the MLA group than the TULAA group. No difference between the two groups was found for the duration of the initial use of antibiotics, interval from initial presentation to operation, or the rates of preoperative percutaneous catheter drainage insertion. Extended resections (especially partial cecectomy) were more frequently performed in the MLA group than in the TULAA group. No difference between the two groups was found for the operative time and open conversion rate. The complication rates were similar in the two groups, but no intra-abdominal fluid collection was detected in the TULAA group. The TULAA group tended to have shorter postoperative hospital stays than the MLA group. However, the total hospital stay was significantly longer in the TULAA group than in the MLA group. None of the patients were re-admitted in either of the two groups.

There were seven (0.91%) unexpected pathologies altogether: A villous adenoma, a carcinoid, and five mucinous cystadenomas.

## 4. Discussion

SLA is a potentially effective alternative to MLA, requiring only a small incision within the umbilical dimpling that was almost invisible postoperatively. However, previous studies, which compared SLA and MLA in adults showed a significantly longer operative time in SLA [7,8,9,10,11,12]. Technical difficulty might explain the longer operative time during the intracorporeal excision of the appendix in SLA. In pediatric surgery, a few studies were on the removal of the appendix through a single umbilical incision and extracorporeal excision [13,14,15,16,17]. TULAA combines the advantages of the favorable intra-abdominal visualization of laparoscopy and the safety and speed of traditional extracorporeal open appendectomy [26]. A few recent reports using TULAA in children showed a significantly shorter operative time than with MLA [18,19,20,21].

In adults, an early study of transumbilical excision of the appendix was reported in 1992 by Pelosi and Pelosi 3rd [27], but it was only a case series of appendectomies for gynecological conditions without a control group, and a conventional laparoscopic cannula was used other than a single port device. However, TULAA is not as widely used in adults as in pediatric surgery. We adopted TULAA in adults and young adolescents, and the results showed a significantly shorter operative time in the TULAA group than in the MLA group, except for the IA subgroup.

The transumbilical excision is technically easier in children than in adults because the distance between the umbilicus and the cecum is shorter in children than in adults, with a more flexible abdominal wall than in adults, facilitating exteriorization of the appendix through the umbilicus [28,29]. Most pediatric studies simply exteriorized the appendix through the umbilicus without dissection of the peritoneal attachments of the appendix and the cecum [15,16,19,21,28,29], although a few studies dissected the peritoneal attachments in some children when needed [17,18,20]. We routinely dissected the peritoneal attachments of the appendix and the cecum in adults in order to exteriorize the appendix easily through the transumbilical incision. We found that it is technically easier to dissect the peritoneal attachments than to ligate the mesoappendix and divide the appendix at its root intracorporeally in the single-port setting.

SLA showed a higher conversion rate than MLA in a recent meta-analysis [12]. In this study, contrarily, no open conversion was detected in the TULAA group, except one case of ileocecal resection for IA. Compared with previous intracorporeal excision, the transumbilical excision of the appendix might prevent open conversion and reduce technical difficulties.

TULAA showed additional advantages, including a lack of postoperative intra-abdominal fluid collection. This could be due to the small sample size and possible selection bias. Other explanations may be a difference of the suction device used. It is easier to remove residual intra-abdominal fluid in TULAA using a suction device for open surgery than in MLA using a laparoscopic suction device. In addition, the postoperative hospital stay was shorter in the TULAA group than in the MLA group, although the difference was small for uncomplicated appendicitis. Lack of postoperative intra-abdominal fluid collection in the TULAA group might have reduced postoperative abdominal pain and shortened the postoperative hospital stay.

The concept of IA began with the idea that the extended resection rate and the complication rate would be lower than for EA [22,24,30], because acute inflammation has already been resolved at the initial conservative treatment in IA, so the peritoneal cavity is not contaminated and peritoneal adhesion is reduced during surgery. Consequently, IA could have some advantages over EA. We found that a relatively lower open conversion rate and a lower complication rate were the advantages of IA. Although the extended resection rate was not significantly different between the EA and IA groups, in the subgroup analysis within the IA group, TULAA had a significantly lower extended resection rate than MLA had. The already resolved inflammation might have resulted in a shorter operative time, a lower open conversion rate, a shorter postoperative hospital stay, and no re-admission in the IA group.

Traditionally, IA has been reported to prevent the recurrence of appendicitis and eliminate the possibility of tumors and inflammatory bowel diseases [31,32]. However, recent studies have suggested that nonoperative treatment is more valuable than IA for perforated appendicitis because no significant difference was found between the complication rate in IA and the recurrence rate in the nonoperative treatment group [22,33,34]. In our findings, the rate of unexpected pathologies and complications was similar to what has been reported in previous studies [22,25]. Unless appendectomy is performed, the recurrence rate of appendicitis ranges from 6% to 20% [33], and patients who do not undergo surgery live with the risk of recurrence throughout their lifetime. When non-operative treatment is chosen for patients after initial conservative treatment, it is impossible to predict the potential risk for patients with hidden tumors.

There are some limitations in our study. First, it was a retrospective study with a small number of patients in a single center. Second, some selection biases exist. The type and timing of the surgeries depended on surgeons’ preferences. In addition, the MLA group showed longer initial, postoperative, and total hospital stays than the TULLA group, which might mean that more complicated patients underwent MLA secondary to selection bias. Actually, we consider it to be a kind of pilot study to find the usefulness and feasibility of TULAA in adults and young adolescents, because it showed some possible advantages over MLA and even over previous SLA. Third, postoperative pain and cosmesis were not investigated.

## 5. Conclusions

TULAA can be a useful surgical alternative to MLA in adults and young adolescents, showing a shorter operative time, a shorter postoperative hospital stay, and rare open conversion. TULAA is feasible for IA in that it showed a lower rate of extended resection and complication. Further larger studies are needed.

## Figures and Tables

**Table 1:** Comparisons between multi-port laparoscopic appendectomy and transumbilical single-incision laparoscopic-assisted appendectomy for uncomplicated appendicitis.

	MLA (*n* = 394)	TULAA (*n* = 46)	*p*-Value
Age (year)	36.3 ± 14.1	39.0 ± 15.0	0.241 ^a^
Sex			0.536
Male	202	26	
Female	192	20	
Body mass index (kg/m^2^)	23.5 ± 3.6	24.4 ± 3.8	0.115 ^a^
Operative time (min)	44.8 ± 16.8	37.2 ± 11.3	<0.001 ^a^
Open conversion	2 (0.5)	0	0.628
Complications	31 (7.8)	5 (10.2)	0.566
Wound infection	22 (5.6)	4 (8.7)	0.335
Ileus	3 (0.8)	1 (2.2)	0.358
Intra-abdominal fluid collection	6 (1.5)	0	1.000
Leakage	0	0	-
Postoperative stay (days)	2.3 ± 0.50	2.1 ± 0.25	<0.001 ^a^
Re-admission	9 (2.3)	1 (2.2)	1.000

Values are expressed as mean ± standard deviation or number (percentage). ^a^ Independent *t* test. MLA: multi-port laparoscopic appendectomy; TULAA: transumbilical single-incision laparoscopic-assisted appendectomy.

**Table 2:** Comparisons between early appendectomy and interval appendectomy for complicated appendicitis.

Variables	EA (*n* = 264)	IA (*n* = 66)	*p*-Value
Age (year)	41.6 ± 15.3	46.8 ± 16.1	0.015 ^a^
Sex Men Women	142 (53.8) 122 (46.2)	29 (43.9) 37 (56.1)	0.152
Body mass index (kg/m^2^)	23.6 ± 3.9	23.5 ± 3.3	0.816 ^a^
Preoperative PCD	n.d.	12 (18.2)	n.a.
Operative approach MLA TULAA	238 (90.2) 26 (9.8)	37 (56.1) 29 (43.9)	<0.001
Extended resections	47 (17.8)	10 (15.2)	0.610
Partial cecectomy	40 (15.2)	6 (9.1)	0.204
Ileocecal resection	7 (2.7)	4 (6.1)	0.168
Operative time (min) MLA TULAA	71.6 ± 37.1 72.6 ± 38.3 62.7 ± 21.4	57.0 ± 41.3 62.5 ± 39.6 54.5 ± 45.9	<0.001 ^b^ 0.017 ^b^ 0.008 ^b^
Open conversion	30 (11.4)	4 (6.1)	0.269
Complications	46 (17.4)	6 (9.1)	0.097
Wound problem	21 (8.0)	2 (3.0)	0.161
Ileus	21 (8.0)	1 (1.5)	0.061
Intraabdominal fluid collection	3 (1.1)	1 (1.5)	0.802
Leakage	1 (0.4)	2 (3.0)	0.042
Postoperative hospital stay (day)	5.6 ± 2.1	4.5 ± 4.3	< 0.001 ^b^
Total hospital stay (day)	5.6 ± 2.1	13.2 ± 11.9	< 0.001 ^b^
Re-admission	8 (3.0)	0	0.152

Values are expressed as mean ± standard deviation or number (percentage). ^a^ Independent *t* test. ^b^ Mann–Whitney U test. EA: early appendectomy; IA: interval appendectomy; PCD: percutaneous catheter drainage; n.d.: no data; n.a.: not applicable; MLA: multi-port laparoscopic appendectomy; TULAA: transumbilical single-incision laparoscopic-assisted appendectomy.

**Table 3:** Comparisons between multi-port laparoscopic appendectomy and transumbilical single-incision laparoscopic-assisted appendectomy within the early appendectomy group for uncomplicated and complicated appendicitis.

Variables	MLA (*n* = 632)	TULAA (*n* = 72)	*p*-Value
Age (year)	38.2 ± 14.8	40.8 ± 14.9	0.130 ^a^
Sex Men Women	329 (52.1) 303 (47.9)	41 (56.9) 31 (43.1)	0.439
Body mass index (kg/m^2^)	23.5 ± 3.7	24.3 ± 3.9	0.092
Complicated appendicitis	238 (37.7)	26 (36.1)	0.790
Extended resections	46 (7.3)	1 (1.4)	0.058
Partial cecectomy	39 (6.2)	1 (1.4)	0.097
Ileocecal resection	7 (1.1)	0	0.369
Operative time (min)	55.3 ± 30.1	46.4 ± 19.9	0.005 ^a^
Open conversion	32 (5.1)	0	0.051
Complications	72 (11.4)	10 (13.9)	0.535
Wound problem	39 (6.2)	8 (11.1)	0.113
Ileus	23 (3.6)	2 (2.8)	0.707
Intraabdominal fluid collection	9 (1.4)	0	0.308
Leakage	1 (0.2)	0	0.736
Postoperative hospital stay (day)	3.6 ± 2.1	3.2 ± 1.5	0.071 ^a^
Re-admission	16 (2.5)	2 (2.8)	0.902

Values are expressed as mean ± standard deviation or number (percentage). ^a^ Mann–Whitney U test. MLA: multi-port laparoscopic appendectomy; TULAA: transumbilical single-incision laparoscopic-assisted appendectomy.

**Table 4:** Comparisons between multi-port laparoscopic appendectomy and transumbilical single-incision laparoscopic-assisted appendectomy within the interval appendectomy group for complicated appendicitis.

Variables	MLA (*n* = 47)	TULAA (*n* = 29)	*p*-Value
Age (year)	48.9 ± 14.9	44.1 ± 17.4	0.237 ^a^
Sex Men Women	15 (40.5) 22 (59.5)	14 (48.3) 15 (51.7)	0.530
Body mass index (kg/m^2^)	23.1 ± 2.9	23.9 ± 3.9	0.303 ^a^
Initial hospital stay (day)	11.0 ± 11.9	6.0 ± 5.0	<0.001 ^b^
Initial use of antibiotics (day)	18.6 ± 11.7	16.7 ± 7.5	0.726 ^b^
Interval from initial presentation to operation (week)	8.1 ± 5.2	7.8 ± 4.7	0.214 ^b^
Preoperative PCD	7 (18.9)	5 (17.2)	0.861
Extended resection	9 (24.3)	1 (3.4)	0.020
Partial cecectomy	6 (16.2)	0	0.024
Ileocecal resection	3 (8.1)	1 (3.4)	0.435
Operative time (min)	61.0 ± 39.0	51.7 ± 44.2	0.114 ^b^
Open conversion	3 (8.1)	1 (3.4)	0.450
Complication	3 (8.1)	3 (10.3)	0.756
Wound problem	1 (2.1)	1(3.4)	0.862
Ileus	0	1 (3.4)	0.259
Intraabdominal fluid collection	1 (2.7)	0	0.376
Leakage	1 (2.7)	1 (3.4)	0.862
Postoperative hospital stay (day)	5.2 ± 4.9	3.5 ± 3.3	0.013 ^b^
Total hospital stay (day)	16.3 ± 13.9	9.5 ± 7.4	< 0.001 ^b^
Re-admission	0	0	n.a.

Values are expressed as mean ± standard deviation or number (percentage). ^a^ Independent *t* test. ^b^ Mann–Whitney U test. MLA: multi-port laparoscopic appendectomy; TULAA: transumbilical single-incision laparoscopic-assisted appendectomy; PCD: percutaneous catheter drainage; n.a.: not applicable.
